# Supplementary material for: Childhood Outcomes in Children with and without Cardiac Echogenic Foci: An Electronic Birth Cohort Study in Wales, UK
Source: Children (Basel). 2023 Jul 17;10(7):1233. doi: 10.3390/children10071233 (PMC10378422; doi:10.3390/children10071233)
Supplement: Supplementary file 1 [file children-10-01233-s001.zip › children-2452009-supplementary.pdf]

Supporting information captions

S1 STROBE checklist

S1 Table. Codes used in the definition of cardiac hospital admissions

S2 Table. Codes used in the definition of other hospital admissions

S1 STROBE Checklist

	Item No	Recommendation	Page in manuscript
**Title and abstract**	1	(*a*) Indicate the study's design with a commonly used term in the title or the abstract	1 (title)<br>2 (abstract)
		(*b*) Provide in the abstract an informative and balanced summary of what was done and what was found	2
**Introduction**			
Background/rationale	2	Explain the scientific background and rationale for the investigation being reported	3-4
Objectives	3	State specific objectives, including any prespecified hypotheses	4
**Methods**			
Study design	4	Present key elements of study design early in the paper	5 onwards
Setting	5	Describe the setting, locations, and relevant dates, including periods of recruitment, exposure, follow-up, and data collection	5
Participants	6	(*a*) Give the eligibility criteria, and the sources and methods of selection of participants. Describe methods of follow-up	5
		(*b*) For matched studies, give matching criteria and number of exposed and unexposed	N/A
Variables	7	Clearly define all outcomes, exposures, predictors, potential confounders, and effect modifiers. Give diagnostic criteria, if applicable	5-8
Data sources/ measurement	8*	For each variable of interest, give sources of data and details of methods of assessment (measurement). Describe comparability of assessment methods if there is more than one group	7-8
Bias	9	Describe any efforts to address potential sources of bias	9-10
Study size	10	Explain how the study size was arrived at	9
Quantitative variables	11	Explain how quantitative variables were handled in the analyses. If applicable, describe which groupings were chosen and why	10
Statistical methods	12	(*a*) Describe all statistical methods, including those used to control for confounding	10
		(*b*) Describe any methods used to examine subgroups and interactions	N/A
		(*c*) Explain how missing data were addressed	10
		(*d*) If applicable, explain how loss to follow-up was addressed	10
		(*e*) Describe any sensitivity analyses	N/A
**Results**			
Participants	13*	(a) Report numbers of individuals at each stage of study—eg numbers potentially eligible, examined for eligibility, confirmed eligible, included in the study, completing follow-up, and analysed	10<br>Figure 1
		(b) Give reasons for non-participation at each stage	10
		(c) Consider use of a flow diagram	Figure 1
Descriptive data	14*	(a) Give characteristics of study participants (eg demographic, clinical, social) and information on exposures and potential confounders	10-11<br>Table 1
		(b) Indicate number of participants with missing data for each variable of interest	Table 1

		(c) Summarise follow-up time (eg, average and total amount)	11
Outcome data	15*	Report numbers of outcome events or summary measures over time	11-13 Tables 2-5
Main results	16	(*a*) Give unadjusted estimates and, if applicable, confounder-adjusted estimates and their precision (eg, 95% confidence interval). Make clear which confounders were adjusted for and why they were included	Tables 2-5
		(*b*) Report category boundaries when continuous variables were categorized	N/A
		(*c*) If relevant, consider translating estimates of relative risk into absolute risk for a meaningful time period	N/A
Other analyses	17	Report other analyses done—eg analyses of subgroups and interactions, and sensitivity analyses	N/A
**Discussion**			
Key results	18	Summarise key results with reference to study objectives	13
Limitations	19	Discuss limitations of the study, taking into account sources of potential bias or imprecision. Discuss both direction and magnitude of any potential bias	14-15
Interpretation	20	Give a cautious overall interpretation of results considering objectives, limitations, multiplicity of analyses, results from similar studies, and other relevant evidence	16
Generalisability	21	Discuss the generalisability (external validity) of the study results	14
**Other information**			
Funding	22	Give the source of funding and the role of the funders for the present study and, if applicable, for the original study on which the present article is based	23

*Give information separately for exposed and unexposed groups.

S1 Table. Codes used in the definition of a cardiac hospital admission

**Primary outcome: An admission for a cardiac cause**
**<u>An admission with an ICD-10 code of</u>**:<br>**I00 – I52** (diseases of the circulatory system relating to the heart)<br>**C38.0** (malignant neoplasm of heart or pericardium)<br>**C38.8** (overlapping neoplasm of heart, mediastinum, or pleura)<br>**D15.1** (benign neoplasm of the heart)<br>**P29** (cardiovascular disorders originating in the perinatal period)<br>**Q20 – Q28** (congenital malformations of the circulatory system)<br><br>This should NOT include:<br>I60 – I69 (cerebrovascular diseases)<br>I70 – I79 (diseases of arteries, arterioles and capillaries)<br>I80 – I89 (diseases of veins, lymphatic vessels, and lymph nodes)<br>I95 – I99 (other and unspecified disorders of the circulatory system)

S2 Table. Codes used in the definition of other hospital admissions

**Secondary outcome: An admission for a respiratory cause**
**An admission with an ICD-10 code of**:<br>**ICD-10:**<br>**J00 – J06** (acute upper respiratory tract infections)<br>**J09 – J18** (influenza and pneumonia)<br>**J20 – J22** (other acute lower respiratory tract infections)<br>**J45 – J46** (asthma)<br>**A37** (whooping cough)<br>**H66 – H67** (otitis media)<br>**P27** (chronic respiratory disease originating in the perinatal period)
**Secondary outcome: An admission for a neoplasm**
**An admission with an ICD-10 code of**:<br>**ICD-10:**<br>**C00 – C97** (malignant neoplasms)<br>**D00 – D48** (in-situ and benign neoplasms)<br><br>Analysed together and separately, with a specific analysis also cnducted to examine admissions for rhabdomyoma (D21.9)
